# The psychosocial determinants of the intention to avoid sexual engagement when intoxicated among young men in KwaZulu-Natal, South Africa

**DOI:** 10.1186/s12889-016-3219-2

**Published:** 2016-07-13

**Authors:** Thabang Manyaapelo, Robert A. C. Ruiter, Anam Nyembezi, Bart van den Borne, Sibusiso Sifunda, Priscilla Reddy

**Affiliations:** Human Sciences Research Council, Population Health, Health Systems and Innovation, Private Bag X41, Pretoria, 0001 South Africa; Department of Work & Social Psychology, Maastricht University, P.O. Box 616, 6200 MD Maastricht, The Netherlands; Human Sciences Research Council, Population Health, Health Systems and Innovation, Private Bag X9182, Cape Town, 8000 South Africa; Department of Health Education & Health Promotion, Maastricht University, P.O. Box 616, 6200 MD Maastricht, The Netherlands; Human Sciences Research Council, HIV/AIDS, STIs and TB, Private Bag X41, Pretoria, 0001 South Africa

**Keywords:** Theory of planned behaviour, HIV/AIDS, Risky sex, Alcohol, Marijuana, Men, South Africa, Responsible manhood

## Abstract

**Background:**

A cross sectional study was conducted among 350 sexually active, mainly unemployed men between the ages of 18 and 35 in KwaZulu-Natal. This study examined the psychosocial determinants of the intention to be sexually active after having used marijuana or alcohol personally or in instances when the sexual partner is intoxicated. The theory of planned behaviour and cultural notions of responsible manhood were used in developing the measures.

**Methods:**

Correlation and hierarchical stepwise linear regression analyses tested determinants of the intention to avoid having sex when personally intoxicated and the intention to avoid sex when the sexual partner is intoxicated.

**Results:**

About 78 % of the participants reported regular use of alcohol and 39 % indicated ever-using marijuana. A total of 36.3 % used both alcohol and marijuana, and 73 % said that they engaged in multiple sexual partner behaviour. The intention to avoid sex when personally intoxicated as well as the intention to avoid sex when the sexual partner is intoxicated were significantly associated with subjective norms and perceptions of perceived behavioural control towards the respective behaviours, and less with attitudes towards the respective behaviours.

**Conclusions:**

These findings imply that health education interventions should focus on changing the normative beliefs as well as control beliefs of the target population either directly through education and training or indirectly by creating physical and social environments that facilitate safe sexual practices, for example by organizing positive peer support for risk prevention and by making condoms freely available in community alcohol serving establishments.

## Background

Alcohol use has been found to be a prominent risk behaviour leading to death and disability globally, and has been linked to non-communicable diseases such as cancer, cardiovascular, and liver disease [1, 2]. In South Africa the average alcohol consumption is reported to be 5 billion litres of alcohol each year, which is about 20 l of alcohol per capita per year, with men generally consuming more volumes than women [3–6]. Furthermore, studies have shown that there is an association between excessive alcohol consumption and an increased sexually transmitted disease acquisition where alcohol is reported to affect behaviour, sexual arousal and is said to also affect the immune system adversely [7, 8]. This association is important in a country like South Africa, which has one the highest prevalence rates of STIs and HIV/AIDS in the world, with many people being in an immune compromised situation [9]. Alcohol use has also been shown as an independent risk factor for intentions to engage in unprotected sex [10]. Because risky sex intentions have been associated with actual risk behaviour, considering alcohol consumption in the context of HIV/STI prevention is of public health importance [10].

In 2012, South Africa counted 6.4 million HIV-infected people, with African females 20–34 years old (31.6 %) and African males 25–49 years old (25.7 %) being the groups with the highest risk [9]. The South African HIV prevalence rate translates into 17 % of the global burden of HIV infection [11]. Studies that explored the association of both alcohol use and marijuana use with risky sexual behaviour found that marijuana and alcohol use are associated with non-communication about sexual risks with sexual partners, with more likelihood to engage in unprotected sex, and a greater propensity to take unmeasured risks [12–15]. A study conducted among black men between the ages of 18 and 45 in South Africa found that men with a history of childhood sexual abuse were more likely to have multiple sexual partners as well as report a greater number of days using marijuana [12]. Furthermore, marijuana and alcohol use have been shown to affect executive control functions such as attention, behavioural flexibility, decision-making, inhibitory control, planning, time estimation and working memory that are crucially involved in top-down control of behaviour such as sexual decision making [16–19].

In light of the above findings, a thorough understanding of the factors that serve as determinants of the use of alcohol and marijuana in relation to risky sexual practices is needed to identify relevant target points for the design of interventions to promote safer sexual practices. In the last three decades, the theory of planned behaviour [20–22], social cognitive theory [23, 24], and protection motivation theory [25] have been the most commonly used frameworks to explain health behaviour (for integrative approaches, see [26, 27]). As conceptual framework for the present study we used the theory of planned behaviour ([28]; for a recent re-formulation see [29]). The theory of planned behaviour proposes that human behaviour is for the most part determined by behavioural intention, which is defined as the motivation to perform a specific behaviour. Intention in turn is determined by attitude, subjective norm, and perceived behavioural control. Attitude represents a person’s evaluation of the anticipated outcomes of the behaviour and is the product of the individual’s behavioural beliefs about the outcomes of performing the behaviour and the evaluation of those behavioural outcomes in terms of importance. Subjective norms are a product of whether an individual believes other meaningful people approve or disapprove of the behaviour and the motivation to comply with the opinions of those people. Lastly, perceived behavioural control is determined by an individual’s beliefs whether there are barriers to their control over the behaviour and the perceived power the individual feels to remove these barriers [28].

The theory of planned behaviour has been used widely to describe health behaviours including drug use, physical activity, dietary behaviours, health screening attendance, and sexual behaviours [30–35]. Attitudes and perceived behavioural control proved to be better predictors of behavioural intentions compared to subjective norms in the majority of these studies, whereas in the African context strong contributions have also been reported for subjective norms [36].

In an attempt to identify a broader range of factors that serve as determinants of substance use, drinking behaviour, and unsafe sex, this study extended the theory of planned behaviour by including measures that relate to the concept of responsible manhood. This is because the study population is drawn from traditional communities in KwaZulu-Natal, a province in South Africa. Within these communities there are traditional notions of responsible manhood, which underpin behaviour. Manhood in the literature is defined in terms of the roles that males play in society [37–39], which is mostly defined against the dominant background of Western culture. Here the contemporary hegemonic masculinity is associated with being white, heterosexual, possessing stereotypical masculine traits of assertiveness, dominance, control, physical strength, and emotional restraint [40–42].

In the traditional African context, manhood has been seen as a systematic socialization process that begins from infancy until adulthood. Some African societies have marked rites of passage like the male and female initiation practices [43–45]. The initiation represents a focal point along the lifelong process of entrenching the mores of a community in rearing the boy and girl child, and may also include reference to sustaining sexual and reproductive health. Beyond initiation practices, teachings of responsible behaviour in young men are continued; for example young men are taught and expected to secure resources for future marriage (called *lobola*) through their own efforts, and in marriage to provide support and security for the family [46]. Concurrently acts of irresponsibility like impregnating girls are strongly discouraged by having the young men pay fines (called *inhlawulo*) should this happen [46]. In developing measures for the responsible manhood concept we draw from the above concepts of providing support and discouraging hurtful interpersonal behaviour.

The aim of this study is to explore the predictors of the intention to avoid sex when the individual is intoxicated and the intention to avoid sex with people who are intoxicated. The objective is to test how these outcome variables are explained first, through their direct proximal variables, which are attitudes, subjective norms and perceived behavioural control towards the respective behaviour of interest, secondly through attitudes, subjective norms, perceived behavioural control and intention towards reducing alcohol and marijuana use, thirdly through beliefs with regard to responsible manhood constructs, and finally through socio-demographic variables which include past substance use.

## Method

### Study design

This paper forms part of a larger behavioural intervention study, which set out to adapt and test an intervention targeting men between the ages of 18 and 35 in KwaZulu-Natal province of South Africa. The intervention aimed to promote the reduction of number of sexual partners, promote condom usage, encourage testing for HIV, encourage more supportive male roles in the community, and lastly discourage use of alcohol and drugs. The paper reports on the baseline data specifically focusing on sexual behaviour collected from the participants prior to administering the curriculum. This study received full ethical clearance from the South African Medical Association Research Ethics Committee (SAMAREC- Protocol MRC 1-09), permission was additionally also granted by the local municipal offices, and the traditional leadership in the areas concerned. Participants gave written informed consent to participate in the study.

### Participants and study setting

The study was conducted in KwaZulu-Natal on the eastern coast of South Africa. It is the second most populated province with 10.8 million people; 86.1 % are African, 7.9 % Indian/Asian, 4.6 % White, and 1.4 % Coloured [47, 48]. The most widely spoken language in KwaZulu-Natal is isiZulu. The inclusion criteria for the study were: male, between the ages of 18 and 35, isiZulu speaking, residing in the area, availability for a follow up in 6 months post intervention. The research participants were recruited from multiple community sites such as schools, churches, and community organizations. Participation in the study was on a voluntary basis to all participants who met the inclusion criteria and were able to take part during the times allocated. Researchers provided transport (where necessary) and lunch to the participants.

The recruitment drive included a well-publicised initiative of talks about the study aims at community meetings, local churches, sports tournaments organised specifically for this purpose, and local community radio stations nearly 12 months before commencing the study. Site A is an urban locality roughly 30 km from Durban with a majority African population while Site B is rural and approximately 250 km from Durban also with a majority African population. A total of 575 young men completed the baseline questionnaire with 350 responding positively to ‘ever having sex’, who were then selected for the purposes of this analysis (*N* = 350). A total of 225 young men who responded negatively to ‘ever having sex’ were excluded.

### Study instruments

Data were collected through an interviewer-administered questionnaire, which took about 2 h to administer with snacks being served in between. This questionnaire was adapted from a previous study among prison inmates’ in KwaZulu-Natal and Mpumalanga [49]. Additionally the content for the questionnaire was derived from a literature review on the topic as well as focus group interviews among the study group. The questionnaire was divided in three sections where the first measured the socio-demographic profile of the participants in terms of age, level of education, employment status and if participant lived alone or not. The second section examined the participant’s sexual risk behaviour in terms of the number of sexual partners and frequency of current behaviours of alcohol and marijuana use. The last section focused on the measurement of the key outcome variables: intention to avoid engaging in sexual activities when one is intoxicated with alcohol or marijuana, and intention to avoid engaging in sexual activities with people who are intoxicated with alcohol or marijuana. In this last section the psychosocial determinants of these outcome variables were also assessed in terms of attitude, subjective norm, and perceived behavioural control towards these behavioural intentions. The questionnaire was developed in English and translated into isiZulu, then it was back translated into English to ensure construct and face validity. The research assistants together with the project managers who came from the same background as the research participants were responsible for the translation process.

### Measures and scale construction

#### Alcohol and marijuana use

Two single items assessed the frequency of alcohol and marijuana use in the past 6 months, respectively, using a 5-point scale with 1 = never (0 days), 2 = rarely (1 to 2 days), 3 = sometimes (3 to 9 days), 4 = often (10 to 19 days), and 5 = very often (20 days or more).

#### Multiple partners

Risky sexual behaviour was measured by asking the number of sexual partners the participant engaged in sex with in the past 6 months. A 4-point scale was used with response options 0 = not sexually active, 1 = 1 sexual partner, 2 = between 2 and 5 sexual partners, 3 = between 6 and 10 sexual partners, and 4 = 10 and more sexual partners.

#### Psychosocial correlates

Using the recommendations from Ajzen on how to measure theory of planned behaviour constructs as a guide [50], measures of attitude, subjective norm, perceived behavioural control, intentions towards behaving as a responsible man, reducing alcohol intake and marijuana use, avoiding engaging in sexual activities when one is intoxicated with alcohol or marijuana, and avoiding engaging in sexual activities with people who are intoxicated with alcohol or marijuana were constructed. The theory of planned behaviour variables (attitudes, subjective norms, perceived behavioural control, and intentions) were measured using a 1 to 5 scale with response options 1 = strongly/fully disagree, 2 = disagree, 3 = unsure, 4 = agree, and 5 = strongly/fully agree, while perceived behavioural control was measured using a 1 to 5 scale with response options 1 = very confident, 2 = confident, 3 = unsure, 4 = not confident, and 5 = not confident at all. Table 1 provides an overview of the psychosocial concepts that were measured, including the number of items, sample items, minimum and maximum score, and using Cronbach’s Alpha (three or more items) or Pearson’s r (two items) as a measure of the internal consistency of grouped items.

**Table 1:** Overview of the scale measures

Measures and example items	Number of items	Min Score	Max Score	Cronbach’s Alpha (α)/Pearson’s r
Attitudes towards behaving as a responsible man.	2	1	5	.59
- A responsible man is someone who has to discipline his wife/partner when necessary using physical force				
- A responsible man is someone who forces himself on his partner when they do not feel like having sex				
Subjective norms towards behaving as a responsible man.	2	1	5	.73
- Most of your community members think that a responsible man is someone who has to discipline his wife/partner when necessary using physical force.				
- Most men I know think that a responsible man is someone who has to discipline his wife/partner when necessary using physical force.				
Perceived Behavioural Control towards behaving as a responsible man.	2	1	5	.42
- How confident are you that you will be able to look after your partner’s wellbeing?				
- How confident are you that you will be able to support your partner and children financially?				
Intentions towards behaving as a responsible man.	1	1	5	-
- I intend to discipline my wife/partner when necessary using physical force in the next 3 months				
Attitudes towards reducing overall alcohol and drug intake	6	1	5	.84
-Reducing overall drug and alcohol intake to only 1 day a week in the next 3 months is something that is wise				
-Reducing overall drug and alcohol intake to only 1 day a week in the next 3 months is something that is pleasant				
-Reducing overall drug and alcohol intake to only 1 day a week in the next 3 months is something that is good				
-Reducing overall drug and alcohol intake to only 1 day a week in the next 3 months is something that is valuable				
-Reducing overall drug and alcohol intake to only 1 day a week in the next 3 months is something that is safe				
-Reducing overall drug and alcohol intake to only 1 day a week in the next 3 months is something that is natural				
Subjective Norms towards reducing overall alcohol and drug intake	5	1	5	.84
-Most people who are important to me think that reducing overall drug and alcohol intake to only 1 day a week in the next 3 months is a good thing				
-Most men who are important to me think that reducing overall drug and alcohol intake to only 1 day a week in the next 3 months is a good thing				
-Most of my peers who are important to me think that reducing overall drug and alcohol intake to only 1 day a week in the next 3 months is a good thing				
-Most community members who are important to me think that reducing overall drug and alcohol intake to only 1 day a week in the next 3 months is a good thing				
-Most family members who are important to me think that reducing overall drug and alcohol intake to only 1 day a week in the next 3 months is a good thing				
Perceived Behavioural Control towards reducing overall alcohol and drug intake	3	1	5	.66
-For me to reduce overall drug and alcohol intake to only 1 day a week in the next 3 months is possible				
- If I wanted to reduce overall drug and alcohol intake to only 1 day a week in the next 3 months I could do it with ease				
- How much control do you believe you have in reducing overall drug and alcohol intake to only 1 day a week in the next 3 months				
Intentions towards reducing overall alcohol and drug intake	6	1	5	.93
-I intend to reduce overall drug and alcohol intake to only 1 day a week in the next 3 months				
-I intend to reduce overall drug and alcohol intake to only 1 day a week in the next 3 months even if I am offered free alcohol and/or drugs				
-I intend to reduce overall drug and alcohol intake to only 1 day a week in the next 3 months even if people are calling me derogatory names				
-I intend to reduce overall drug and alcohol intake to only 1 day a week in the next 3 months even if i acquire material wealth				
-I intend to reduce overall drug and alcohol intake to only 1 day a week in the next 3 months even if my friends dare me				
-I intend to reduce overall drug and alcohol intake to only 1 day a week in the next 3 months even if my drinking partners dare me				
Attitudes towards avoiding sex when you are intoxicated	5	1	5	.91
-Avoiding engaging in sex when under the influence of drugs or alcohol in the next 3 months is something that is wise				
-Avoiding engaging in sex when under the influence of drugs or alcohol in the next 3 months is something that is pleasant				
-Avoiding engaging in sex when under the influence of drugs or alcohol in the next 3 months is something that is good				
-Avoiding engaging in sex when under the influence of drugs or alcohol in the next 3 months is something that is valuable				
-Avoiding engaging in sex when under the influence of drugs or alcohol in the next 3 months is something that is safe				
Subjective Norms towards avoiding sex when you are intoxicated	5	1	5	.84
-Most people who are important to me think that avoiding engaging in sex when under the influence of drugs or alcohol in the next 3 months is a good thing				
-Most men who are important to me think that avoiding engaging in sex when under the influence of drugs or alcohol in the next 3 months is a good thing				
-Most of my peers who are important to me think that avoiding engaging in sex when under the influence of drugs or alcohol in the next 3 months is a good thing				
-Most community members who are important to me think that avoiding engaging in sex when under the influence of drugs or alcohol in the next 3 months is a good thing				
-Most family members who are important to me think that avoiding engaging in sex when under the influence of drugs or alcohol in the next 3 months is a good thing				
Perceived Behavioural Control towards avoiding sex when you are intoxicated	3	1	5	.66
- For me to avoid engaging in sex when under the influence of drugs or alcohol in the next 3 months is possible				
- If I wanted to avoid engaging in sex when under the influence of drugs or alcohol in the next 3 months I could do it with ease				
- How much control do you believe you have in avoid engaging in sex when under the influence of drugs or alcohol in the next 3 months				
Intentions towards avoiding sex when you are intoxicated	6	1	5	.92
- I intend to avoid engaging in sex when under the influence of drugs or alcohol in the next 3 months				
- I intend to avoid engaging in sex when under the influence of drugs or alcohol in the next 3 months even if I am offered free alcohol and/or drugs				
- I intend to avoid engaging in sex when under the influence of drugs or alcohol in the next 3 months even if people are calling me derogatory names				
- I intend to avoid engaging in sex when under the influence of drugs or alcohol in the next 3 months even if I acquire material wealth				
- I intend to avoid engaging in sex when under the influence of drugs or alcohol in the next 3 months even if my friends dare me				
- I intend to avoid engaging in sex when under the influence of drugs or alcohol in the next 3 months even if my drinking partners dare me				
Attitudes towards avoiding sex with people who are intoxicated	5	1	5	.94
-Avoiding engaging in sex with people who are under the influence of drugs or alcohol in the next 3 months is something that is wise				
-Avoiding engaging in sex with people who are under the influence of drugs or alcohol in the next 3 months is something that is pleasant				
-Avoiding engaging in sex with people who are under the influence of drugs or alcohol in the next 3 months is something that is good				
-Avoiding engaging in sex with people who are under the influence of drugs or alcohol in the next 3 months is something that is valuable				
-Avoiding engaging in sex with people who are under the influence of drugs or alcohol in the next 3 months is something that is safe				
Subjective Norms towards avoiding sex with people who are intoxicated	5	1	5	.88
-Most people who are important to me think that avoiding engaging in sex with people who are under the influence of drugs or alcohol in the next 3 months is a good thing				
-Most men who are important to me think that avoiding engaging in sex with people who are under the influence of drugs or alcohol in the next 3 months is a good thing				
-Most of my peers who are important to me think that avoiding engaging in sex with people who are under the influence of drugs or alcohol in the next 3 months is a good thing				
-Most community members who are important to me think that avoiding engaging in sex with people who are under the influence of drugs or alcohol in the next 3 months is a good thing				
-Most family members who are important to me think that avoiding engaging in sex with people who are under the influence of drugs or alcohol in the next 3 months is a good thing				
Perceived Behavioural Control towards avoiding sex with people who are intoxicated	3	1	5	.79
- For me to avoid engaging in sex with people who are under the influence of drugs or alcohol in the next 3 months is possible				
- If I wanted to avoid engaging in sex with people who are under the influence of alcohol in the next 3 months I could do it with ease				
- How much control do you believe you have in avoid engaging in sex with people who are under the influence of alcohol in the next 3 months				
Intentions towards avoiding sex with people who are intoxicated	6	1	5	.93
- I intend to avoid engaging in sex with people who are under the influence of drugs or alcohol in the next 3 months				
- I intend to avoid engaging in sex with people who are under the influence of drugs or alcohol in the next 3 months even if I am offered free alcohol and/or drugs				
- I intend to avoid engaging in sex with people who are under the influence of drugs or alcohol in the next 3 months even if people are calling me derogatory names				
- I intend to avoid engaging in sex with people who are under the influence of drugs or alcohol in the next 3 months even if I acquire material wealth				
- I intend to avoid engaging in sex with people who are under the influence of drugs or alcohol in the next 3 months even if my friends dare me				
- I intend to avoid engaging in sex with people who are under the influence of drugs or alcohol in the next 3 months even if my drinking partners dare me				

### Analysis

Statistical analysis was done using SPSS Version 22. Descriptive statistics were used to describe the sample. Bivariate correlations analysis was used to assess associations between study measures. Hierarchical linear regression models were then used to determine the unique contribution the study measures made to explaining the overall variance in firstly the intention to avoid sex when intoxicated and secondly the intention to avoid sex with people who are intoxicated. The stepwise regression was done in a four-step process starting with the more proximal and ending with more distal predictors. In step 1 the outcome variable is tested against the proximal predictors attitude, subjective norm and perceived behavioural control towards the behaviour of interest. In step 2 attitudes, subjective norm, perceived behavioural control, and intention towards reducing alcohol and marijuana used were added. Step 3 added attitude, subjective norm, perceived behavioural control, and intention towards responsible manhood. Finally, the socio-demographic variables (age, level of education) and behavioural variables (past substance use and sexual behaviour) were added in step 4.

## Results

### Socio-demographic profile of the participants

A total of 350 young men were included in the analysis. The ages ranged from 18 to 35 with the majority (64.6 %) between the ages of 18 and 20 (see Table 2). The level of education for this sample varied from primary education to tertiary education with 40 % (*N* = 143) of the sample having a grade 12 and higher qualification. Almost all participants reported being unemployed (95.7 %). Most participants lived with at least one parent or with a relative.

**Table 2:** Socio-demographic profile of the participants

*Characteristic*	*Frequency*	*Percentage*
*Age*		
*(18–20)*	*226*	*64.6 %*
*(21–25)*	*85*	*24.3 %*
*(26–30)*	*30*	*8.6 %*
*(31–35)*	*7*	*2.0 %*
*Levels of education*		
*Primary*	*1*	*.3 %*
*Standard 6*	*33*	*9.4 %*
*Standard 8*	*156*	*44.6 %*
*Matric*	*100*	*28.6 %*
*Tertiary (Technikon)*	*25*	*7.1 %*
*Tertiary (University)*	*18*	*5.1 %*
*No formal education*	*0*	*0 %*
*Participants not employed*	*335*	*95.7 %*
*Participants living on their own*	*34*	*9.7 %*
*Participants living with at least one parent*	*108*	*30.8 %*
*Participants living with a relative*	*42*	*12 %*

### Substance use and sexual behaviours

Just over three quarters (78.3 %) reported having used alcohol before and 38.9 % ever used marijuana. A total of 36.3 % of the participants used both alcohol and marijuana. About 73 % reported having multiple concurrent sexual partnerships.

#### Predictors of intention to avoid sex when intoxicated

Table 3 presents the correlations as well as the results of the hierarchical stepwise analysis assessing the unique contributions of the predictor variables in the explanation of intention to avoid sex when one is under the influence of alcohol and marijuana.

**Table 3:** Hierarchical regression of the intention to avoid sex when intoxicated

		Step1		Step 2		Step 3		Step 4	
	r	b	β	b	β	b	β	b	β
*Avoid sex when intoxicated*									
Attitude	.227**	-.00	-.00	-.07	-.09	-.06	-.08	-.07	-.08
Subjective norm	.460**	.37	.38***	.19	.20***	.20	.20***	.20	.20***
Perceived behavioural control	.312**	.21	.19***	.20	.18***	.20	.18***	.19	.17***
*Reduce alcohol and marijuana use*									
Attitude	.226**			.08	.07	.07	.07	.07	.06
Subjective norm	.492**			.15	.14***	.14	.13**	.13	.13**
Perceived behavioural control	.171**			-.04	-.03	-.04	-.03	-.04	.03
Intention	.580**			.40	.39***	.41	.40***	.41	.39***
*Responsible manhood*									
Attitude	.107*					.03	.05	.03	.05
Subjective norm	.011					.03	.06	.03	.07
Perceived behavioural control	.122*					.00	.13	.00	.00
Intention	.145**					-.00	-.00	.00	.00
Demographic and past behaviour									
Age	-.048							-.10	-.07
Level of education	.035							.04	.04
Relationship status	-.095							-.07	-.03
Marijuana use	.027							.00	.00
Alcohol use	-.059							-.02	-.01
Number sex partners	-.047							.02	.01
Constant		1.79		0.36		−0.11		0.04	
Adjusted R Square		.22***		.42***		.42		.41	

Note: ***p <.05, ** *p* <. 01, *p <.05; b = unstandardized coefficient, β = standardised Beta, *N* = 350

Bivariate correlation analyses were conducted to assess the strength of associations between the outcome variables and the psychosocial measures with *r* = .10 -.23 indicating a small effect, *r* = .24 -.36 indicating a moderate effect, and *r* >.37 indicating a large effect [51, 52]. Strong positive associations with intention to avoid sex when intoxicated were found for subjective norms towards avoiding sex when intoxicated and for intention and subjective norms towards reducing alcohol and marijuana use. Perceived behavioural control towards avoiding sex when intoxicated was found to be moderately associated with the intention to avoid sex when intoxicated. Weak associations were found for attitudes towards avoiding sex when intoxicated, attitudes and perceived behavioural control towards reducing alcohol and marijuana use, and also for attitudes, perceived behavioural control and intention towards behaving as a responsible man.

In the first step of the multiple regression analysis, attitude, subjective norm and perceived behavioural control towards avoiding sex when one is intoxicated explained 22 % of the variance in intention, showing significant contributions of subjective norm and perceived behavioural control (*p* <.001). Adding the psychosocial measures with regard to reducing alcohol and marijuana use in the second step explained an additional 20 % of variance (*p* ≤.001), with significant additional contributions of subjective norm and intentions towards reducing alcohol and marijuana use. The third step added the responsible manhood variables, but without explaining any additional variance. Finally, in the fourth step adding socio-demographic variables, past alcohol and marijuana use explained also no additional variance. The final model explained 41 % of the total variance in the intention to avoid sex when one is intoxicated with significant unique positive contributions of subjective norms and perceived behavioural control to avoid sex when intoxicated as well as the subjective norms and intentions to reduce alcohol and marijuana use.

#### Predictors of intention to avoid sex with people who are intoxicated

Table 4 presents the correlations as well as the results of the hierarchical stepwise analysis including the predictors of intention to avoid sex with people who are under the influence of alcohol and marijuana.

**Table 4:** Hierarchical regression of the intention to avoid sex with people who are intoxicated

		Step1		Step 2		Step 3		Step 4	
	r	b	β	b	β	b	β	b	β
*Avoid sex intoxicated people*									
Attitude	.167**	-.01	-.01	-.02	-.03	-.02	-.02	-.02	-.02
Subjective norm	.413**	.30	.31***	.17	.18***	.16	.17***	.16	.17***
Perceived behavioural control	.213**	.17	.19***	.13	.14***	.12	.13***	.12	.14***
*Reduce alcohol and marijuana use*									
Attitude	.290**			.17	.16***	.18	.17***	.16	.15***
Subjective norm	.378**			.15	.14***	.14	.13***	.15	.13***
Perceived behavioural control	.114*			-.11	-.09	-.12	-.10	-.12	-.09
Intention	.479**			.34	.33***	.35	.34***	.35	.33***
*Responsible manhood*									
Attitude	.125*					.04	.08	.04	.08
Subjective norm	.060					.03	.06	.03	.06
Perceived behavioural control	.118*					.02	.05	.02	.04
Intention	.100					-.01	-.01	.01	-.01
Demographic and past behaviour									
Age	.014							-.01	.00
Level of education	.075							.05	.05
Relationship status	-.066							-.01	.00
Marijuana use	.018							.04	-.06
Alcohol use	-.076							-.01	-.01
Number sex partners	.008							-.00	-.00
Constant		2.35		7.35		.06		.07	
Adjusted R Square		.17***		.37***		.37		.37	

Note: ***p <.05, ** *p* <. 01, *p <.05; b = unstandardized coefficient, β = standardised Beta, *N* = 350

The intention to avoid sex with people who are intoxicated showed strong positive correlations with subjective norms towards avoiding sex with people who are under the influence of alcohol and marijuana and also with both subjective norms and intention towards reducing alcohol and marijuana use. Attitude towards reducing alcohol and marijuana use correlated moderately with the intention to avoid sex with people who are intoxicated. Weak correlations were found for attitude and perceived behavioural control towards avoiding sex with people who are intoxicated, perceived behavioural control towards reducing alcohol and marijuana use, and for attitudes and perceived behavioural control towards behaving as a responsible man.

In the first step of the multiple regression analysis, attitude, subjective norm, and perceived behavioural control explained 17 % of the variance in the intention to avoid sex with people who are intoxicated, showing significant contributions of subjective norm and perceived behavioural control. Adding the variables in the second step explained an additional 20 % of variance (*p* ≤.001), with contributions of attitudes, subjective norms and intentions towards reducing alcohol and marijuana use. The third step added the responsible manhood variables but these variables did not explain additional variance. Finally, in the fourth step adding demographic variables and past alcohol and marijuana use also explained no additional variance. The final model explains 37 % of the variance in the intention to avoid sex with people who are intoxicated with unique significant contributions of subjective norms and perceived behavioural control to avoid sex with people who are intoxicated as well as the attitudes, subjective norms and intentions to reduce alcohol and marijuana use.

## Discussion

The results of this study highlight some important aspects about substance use and sexual behaviours that put young men and their sexual partners at risk of STIs including HIV. Alcohol use was reported by over three quarters of the participants and over one third of the men indicated ever using marijuana. While the rate of alcohol use is higher compared to the most recent national survey among school going youth in South Africa where 49.2 % reported to ever had one or more drinks of alcohol in their lifetime [53], the reported marijuana use at 39.9 % is similar to the findings in the current study.

The purpose of this study was to explore the determinants of the intention to avoid sex when one is under the influence of alcohol and marijuana and the intention to personally avoid sex with people who are under the influence of alcohol and marijuana. This was done using constructs from the theory of planned behaviour. The overall explained variance for both outcome variables ranged between 17 and 42 %, which confirms results from earlier studies that use the theory of planned behaviour to explore health behaviour motivation [30]. It was found that attitude, subjective norm and perceived behavioural control towards avoiding sex when one is intoxicated explained 22 % of the variance in intention, showing significant contributions of subjective norm and perceived behavioural control. For the intention to avoid sex with people who are intoxicated, attitude, subjective norm, and perceived behavioural control explained 17 % of the variance in intention showing again significant contributions of subjective norm and perceived behavioural control, and not attitude. These findings confirm earlier research that societal pressures can have a strong influence on individual behaviour, next to evaluations of behavioural outcomes and feelings of confidence in executing the behaviour [36, 54, 55].

In addition, we were also interested in exploring how sexual decision-making is affected by perceptions of alcohol and marijuana use. Looking at the intention to avoid sex when one is intoxicated we found that the psychosocial measures with regard to reducing alcohol and marijuana use explained an additional 20 % of the variance, with significant additional contributions of subjective norm and intentions towards reducing alcohol and marijuana use. Similarly adding the measures relating to reducing alcohol and marijuana use to the intention to avoid sex with people who are intoxicated also explained an additional 20 % but here with significant additional contributions of attitudes, subjective norms and intentions towards reducing alcohol and marijuana use.

This study also sought to introduce the construct of responsible manhood, which we thought would explain part of the behavioural intention to have safe sex. However the responsible manhood variables emphasizing supportive and non-hurtful behaviour towards partners did not explain any additional variance for both the intention to avoid sex when intoxicated and the intention to avoid sex with people who are intoxicated. Although the correlation associations of the responsible manhood constructs with both intention measures were positive, they were also weak.

Finally, the socio-demographic variables age, level of education, relationship status, number of sexual partners and past use of alcohol and marijuana did not explain any additional variance as would be expected by using the theory of planned behaviour in which person characteristics determine intention and behaviour through the beliefs underlying the general evaluations of behavioural outcomes, social norms, and personal capabilities.

The strong associations of subjective norms with the outcome measures in this study suggest that the men sampled are strongly influenced by their peers and communities with regards to decisions about whether to drink alcohol or smoke marijuana and also in relation to intentions to avoid risky sexual behaviours. Participants in this study do acknowledge the negative behaviours as evidenced in the positive intentions towards avoiding sex when intoxicated and the positive intentions towards avoiding sex with people who are intoxicated, but are weighed on heavily by what their community, friends and people important to them think about these behaviours. The participants engaged in these risky sexual behaviours even though they had strong intentions to avoid them.

The findings suggest that people with a more positive motivation to reduce substance use are more likely to avoid risky sexual practices. As a result, the focus of interventions can be two fold by targeting both the normative beliefs (subjective norms) and control beliefs (perceived behavioural control) towards avoiding risky sexual behaviour and the psychosocial correlates towards reduction of alcohol and marijuana use. Health education interventions should therefore focus on changing the normative beliefs of the target population to create positive social norms towards safe sexual practices in relation to substance use e.g. by showing positive peer support for risk prevention and by building personal resistance against social pressures for risky taking behaviour, and by enabling physical as well as social environments towards safe sexual practices in the context of substance use. A practical intervention enabling positive physical environments in the context of substance use could be to place messages promoting safe sex in community alcohol serving establishments and place free condoms at such venues. This can be done utilising the Intervention Mapping approach, which is a protocol for developing theory and evidence based behaviour change interventions. There are six steps, where each is made up of tasks that incorporate theory and evidence. The completion of each step serves as a guide for the subsequent step, whereby when all the steps are completed the result is a blueprint for designing, implementing and evaluating an intervention based on a foundation of theoretical, empirical, and practical information [56].

Future research, in addition to exploring the impact of subjective norms as a predictor of behavioural intention should closely examine the behaviour of the participant’s peers and close family to establish the degree to which these perceptions influence their intentions.

The present study is not without limitations. Foremost, the responsible manhood concept should be investigated and developed further. Recent research shows how the responsible manhood concept is positively affiliated to ethnic identity and how in turn ethnic identity was shown to have a positive association with safer sexual practices [44]. A potential reason why this study was unable to show strong correlations with the responsible manhood constructs may be due to the poor construction of the variables making them unable to thoroughly test what had been intended. The construction could have emphasised mainly on linking responsible manhood to ethnic identity. As this concept is still in the development stages, better measurement tools should be developed for future studies.

The results showed that about a third of men in the study engage in risky sexual behaviour and substance use. These figures may well be inflated due to the large number of unemployed men sampled. This bias could have inadvertently been as a result of the inclusion criteria, which restricted availability of the potential participants to working hours during the week (08h00 to 17h00, Monday to Fridays).

The limitations notwithstanding, this current study was able to explain a significant proportion of the variance in intentions to avoid sex when intoxicated and when the sexual partner is intoxicated. The findings suggest that health education interventions should focus on changing the normative beliefs as well as control beliefs of the target population either directly through education and training or indirectly by creating more positive social norms and enabling physical environments towards safe sexual practices in the context of substance use. The normative beliefs are especially relevant in Africa where the socialisation of the individual is largely community orientated as evident in extended families, which still dominate the mainly rural landscape.

## Conclusions

The results of this study highlight some important aspects about substance use and sexual behaviours that put young men and their sexual partners at risk of STIs including HIV. The predictors identified by this study reveal opportunities for the development of health education to promote safer sex among men in KwaZulu-Natal Province. These findings imply that health education interventions should focus on changing the normative beliefs as well as control beliefs of the target population either directly through education and training or indirectly by creating physical and social environments that facilitate safer sexual practices, for example by organizing positive peer support for risk prevention and by making condoms freely available in community alcohol serving establishments.

## Abbreviations

AIDS, acquired immune deficiency syndrome; HIV, human immunodeficiency virus; HSRC, Human Sciences Research Council (South Africa); MRC, Medical Research Council of South Africa; SAMAREC, South African Medical Association Research Ethics Committee; STI, sexual transmitted infection; TB, tuberculosis
